# Mini-*α*A Upregulates the miR-155-5p Target Gene CDK2 and Plays an Antiapoptotic Role in Retinal Pigment Epithelial Cells during Oxidative Stress

**DOI:** 10.1155/2023/6713094

**Published:** 2023-02-14

**Authors:** Qianyin Chen, Huimin Lin, Shengnan Li, Xuan Deng, Jinglin Zhang

**Affiliations:** ^1^Guangzhou Aier Eye Hospital, Jinan University, Guangzhou 510040, China; ^2^Sichuan Eye Hospital, Aier Eye Hospital Group, Chengdu 610047, China; ^3^The First Affiliated Hospital of Chongqing Medical University, Chongqing 420030, China; ^4^Aier School of Ophthalmology, Central South University, Changsha 410083, China

## Abstract

**Background:**

Age-related macular degeneration (AMD) is the leading cause of serious vision loss in the elderly. Regulating microRNA (miRNA) gene expression offers exciting new avenues for treating AMD. This study aimed to investigate whether miRNAs and their target genes play an antiapoptotic role during oxidative stress-induced apoptosis of retinal pigment epithelial (RPE) cells via mini-*α*A.

**Methods:**

ARPE-19 cells were treated with 3.5 mM NaIO3 for 48 h to establish a retinal degeneration model. Cells were treated with mini-*α*A (10, 15, and 20 *μ*M) for 4 h. miR-155-5p was knocked down and overexpressed. Cell viability and apoptosis were measured using the Cell Counting Kit-8 assay and flow cytometry, respectively. The reactive oxygen species level was detected by flow cytometry. miR-155-5p target genes were predicted via bioinformatics. Gene Ontology and Kyoto Encyclopedia of Genes and Genomes analyses were performed for miR-155-5p target genes. A quantitative real-time polymerase chain reaction was performed to detect miRNAs and cell cycle-related target genes. Western blotting was performed to measure the levels of apoptotic pathway genes encoding Bcl-2, Bax, cleaved caspase-3, and cyclin-dependent kinase 2 (CDK2). Dual-luciferase reporter gene assay was performed to verify the targeted binding relationship between miR-155-5p and CDK2.

**Results:**

NaIO3 can induce oxidative damage and promote apoptosis. Conversely, mini-*α*A had inhibitory effects and could reverse the oxidative damage and apoptosis triggered by NaIO3 in the retinal degeneration model. The expression of miR-155-5p was upregulated in cells treated with NaIO3 and was downregulated after mini-*α*A treatment. Furthermore, miR-155-5p can target the following cell cycle-related and proliferation-related genes: CDK2, CDK4, CCND1, and CCND2. Moreover, our study indicated that miR-155-5p was involved in the antioxidative damage and antiapoptotic effects of mini-*α*A via CDK2 regulation.

**Conclusions:**

miR-155-5p promotes the antioxidative damage and antiapoptotic effects of mini-*α*A during oxidative stress-induced apoptosis of RPE cells via CDK2 regulation. This study provides a new therapeutic target for AMD.

## 1. Introduction

Retinal pigment epithelial (RPE) cells form a layer between the extracellular segments of the choroid and photoreceptors, constituting a key structure for maintaining normal retinal metabolism and visual function [1]. The abnormal function and apoptosis of RPE cells caused by aging, injury, metabolism, and genetic abnormalities can lead to retinal degeneration, visual impairment, and even irreversible vision loss, representing an important class of blinding diseases, including age-related macular degeneration (AMD), retinitis, and Stargardt's macular dystrophy [2]. The global prevalence of AMD has reached 8.7%, representing one of the most important causes of blindness among the elderly worldwide [3, 4]. AMD is clinically divided into early (moderate retinal edema and retinal pigment changes) and late (neovascularization and atrophy) stages [5]. The pathogenesis of AMD involves the interaction between genetic polymorphisms and environmental risk factors, many of which lead to an increased oxidative stress in the retina [6]. In recent years, although gene and stem cell therapies have brought hope to patients with such diseases, most patients, especially those in the early stage of AMD, cannot benefit from these novel therapies owing to ethical problems, treatment risks, timing, efficacy, and high cost. RPE cell apoptosis is involved in the coinitiation mechanism of AMD and occurs throughout disease progression [7]. Therefore, it is of great clinical value to investigate the mechanism of maintaining the function and homeostasis of RPE cells and inhibiting apoptosis in these cells, leading to new therapeutic avenues.

Increasing evidence suggests that regulating microRNA (miRNA) expression provides exciting new avenues for the research and treatment of AMD [8]. Dysregulation of miR-17, miR-125b, and miR-155 has been reported in various mouse models of AMD as well as in the plasma and retina of individuals with AMD [9]. SanGiovanni et al. reported that miR-155-5p expression was significantly upregulated in the advanced AMD retina [10]. In addition, dysregulation of miR-9, miR-34a, and miR-155 has been reported in the serum of patients with AMD [11]. Therefore, miRNAs are potential biomarkers and novel pharmacological targets for AMD. Hou et al. showed that miRNA-34a inhibited RPE cell proliferation and migration by downregulating its target cyclin-dependent kinases (CDK) 2 and 6 and other cell cycle-related molecules [12]. Through bioinformatics analysis, we revealed that miR-155-5p potentially targets the cell cycle- and proliferation-related genes encoding CDK2, CDK4, cyclin D1 (CCND1), and cyclin D2 (CCND2). This emphasizes the need to further explore the role of miR-155-5p and its downstream target genes in the development and progression of AMD.

*α*-Crystallins (*α*A and *α*B) and their derivatives have received increasing attention due to their great potential in preventing cell death [13]. Recent studies have reported that the expression of *α*A- and *α*B-crystallin is significantly upregulated in the cytosol and mitochondria of RPE cells in light-induced injury, retinal trauma, and other models of acute retinal degeneration. Moreover, the administration of human *α*A- or *α*B-crystallin protects RPE cells from oxidative and endoplasmic reticulum stress-induced apoptosis [14]. Several studies have confirmed that decreased *α*-crystallin expression can increase oxidative stress-induced cell death sensitivity, whereas increased *α*-crystallin expression exerts a protective effect [15–17]. A previous study revealed that the antiapoptotic effect exerted by *α*A-crystallin is associated with its molecular chaperone activity [14]. mini-*α*A is a functional fragment of *α*A-crystallin with molecular chaperone activity [18] and inhibits caspase-3 activation, thus protecting RPE cells from oxidative stress-induced apoptosis [19]. A previous study revealed that mini-*α*A can reduce apoptosis induced by NaIO_3_ in RPE cells, thus exerting protective effects during retinal degeneration [20]. However, its specific mechanism of action remains unclear. Therefore, identifying novel regulators mediated by mini-*α*A may help understand the molecular mechanisms of the antiapoptotic effects of mini-*α*A in RPE cells.

This study used mini-*α*A to treat a NaIO_3_-induced retinal degeneration model and evaluate its therapeutic effects. Through bioinformatics prediction and validation, we further revealed the antiapoptotic effects of mini-*α*A on oxidative stress-induced apoptosis in RPE cells, ultimately providing a new therapeutic target for AMD.

## 2. Materials and Methods

### 2.1. Cell Culture and Treatment

Human ARPE-19 cells were purchased from Cellcook (#CC4001; Guangzhou, China) and cultured in Dulbecco's modified Eagle medium/Nutrient Mixture F-12 (C11765500BT; Gibco) containing 10% fetal bovine serum (C38010050; BI). ARPE-19 cells were seeded in 6-well plates at a concentration of 6 × 10^5^ cells/well and exposed to 3.5 mM NaIO_3_ (S4007; Sigma) for 48 h to establish a retinal degeneration model [21]. mini-*α*A (RP21154; Genscript) was added to the cells at different concentrations (10, 15, and 20 *μ*M) for 4 h [22, 23] to obtain the following control and experimental groups: control, NaIO_3_, NaIO_3_ + 10 *μ*M mini-*α*A, NaIO_3_ + 15 *μ*M mini-*α*A, and NaIO_3_ + 20 *μ*M mini-*α*A. Following the experimental screening, 10 *μ*M mini-*α*A was selected for subsequent experiments in the following subgroups: ARPE-19-negative control (NC), ARPE-19-NC + NaIO_3_, and ARPE-19 + NaIO_3_ + mini-*α*A.

### 2.2. Cell Transfection

To determine whether miR-155-5p is involved in the antioxidative damage effect of mini-*α*A, miR-155-5p inhibitors and mimics were used to interfere and overexpress miR-155-5p. miR-155-5p inhibitor and mimics were transfected in ARPE-19 cells at a final concentration of 100 nM for 48 h to obtain the following experimental groups: NaIO_3_ + NC inhibitor, NaIO_3_ + miR-155-5p inhibitor, NaIO_3_ + NC mimics, NaIO_3_ + mini-*α*A + NC mimics, and NaIO_3_ + mini-*α*A + miR-155-5p mimics. miR-155-5p inhibitor, NC inhibitor, miR-155-5p mimics, and NC mimics were designed and synthesized by GenePharma (Shanghai, China).

### 2.3. Cell Counting Kit-8 (CCK-8) Assay

ARPE-19 cells were cultured in 96-well plates (10000 cells/well) and allowed to adhere overnight under 5% CO_2_ at 37°C. Then, cells were divided into different groups and exposed to different treatments. Finally, 10 *μ*L of CCK-8 reagent (C0040; Beyotime, China) was added to each well for 2 h. Absorbance (450 nm) was measured using a microplate reader (Infinite M200; Tecan, Austria) to determine cell viability at 24, 48, and 72 h.

### 2.4. Reactive Oxygen Species (ROS) Detection

The ROS level was measured using an ROS assay kit (S0033S; Beyotime). 2′,7′-Dichlorodihydrofluorescein diacetate (DCFH-DA; stock concentration, 10 mM) was diluted at 1 : 1000 in a serum-free medium to a final concentration of 10 *μ*M. The treated cells (mentioned above) were removed from the cell culture medium, and DCFH-DA was added to cover the cells. Subsequently, the cells were again incubated at 37°C for 20 min. The samples were collected by trypsinization and flow cytometry (A00-1-1102; Beckman, USA) detection was performed.

### 2.5. Cell Apoptosis Assay

ARPE-19 cells were digested with trypsin (without EDTA). The trypsinized cells were then washed twice with phosphate-buffered saline and centrifuged at 2000 rpm for 5 min. Next, 500 *μ*L of binding buffer was added to the cells in suspension, followed by the addition and thorough mixing of 5 *μ*L of annexin V-allophycocyanin (KGA1022; KeyGen, China) and 5 *μ*L of 7-AAD (00-6993-50; Invitrogen, USA). After incubation for 15 min at room temperature under dark conditions, the apoptosis rate was measured using flow cytometry within 1 h (A00-1-1102; Beckman).

### 2.6. Quantitative Real-Time Polymerase Chain Reaction (qRT-PCR)

The expression of miR-9-5p, miR-125b-5p, miR-34a-5p, miR-184, miR-155-5p, miR-3131, miR-4497, miR-4491, CDK2, CDK4, CCND1, and CCND2 was measured using qRT-PCR. Briefly, total RNA was extracted using Trizol reagent, followed by cDNA preparation using a reverse transcription kit (#CW2569; Beijing ComWin Biotech, China). UltraSYBR Mixture (#CW2601; Beijing ComWin Biotech) was added to determine the relative gene expression using ABI 7900 System. Using glyceraldehyde 3-phosphate dehydrogenase (GAPDH) or U6 as internal controls, the relative gene expression was calculated via the 2^−ΔΔCt^ method.  Table 1 lists the primer sequences used in this study.

### 2.7. Western Blotting

RIPA lysis buffer (#P0013B; Beyotime) was used to extract total protein from cells according to the manufacturer's protocol. Protein was quantified using a BCA protein determination kit (#23225, Thermo Fisher Scientific, USA). Following the addition of loading buffer to the protein samples, the mixture was kept in water at 100°C for 5 min and sodium dodecyl sulfate-polyacrylamide gel electrophoresis was performed. Protein samples were then transferred to a polyvinylidene fluoride membrane. This membrane was sealed with 5% skim milk and blocked for 2 h at room temperature. Subsequently, it was incubated overnight at 4°C with the following primary antibodies: Bcl-2 (3498; CST), Bax (50599-2-Ig; Proteintech), cleaved caspase-3 (9664; CST), CDK2 (2546; CST), and GAPDH (60004-1-Ig; Proteintech). Peroxidase-AffiniPure goat anti-rabbit IgG (*H* + *L*; 111-035-003; Jackson) and peroxidase-AffiniPure goat anti-mouse IgG (*H* + *L*; 115-035-003; Jackson) were used as secondary antibodies. For enhanced chemiluminescence, Odyssey Infrared Imaging System (LI-COR Biosciences, Lincoln, NE, USA) was used to detect the proteins, with GAPDH as the internal reference.

### 2.8. Screening and Bioinformatics Prediction of miRNAs

Eight miRNAs (miR-9-5p, miR-125b-5p, miR-34a-5p, miR-184, miR-155-5p, miR-3131, miR-4497, and miR-4491) were screened for verification [9–11]. Analysis using multiple tools (miRPathDB, https://mpd.bioinf.uni-sb.de/mirna.html?mirna=hsa-miR-155-5p&organism=hsa, hg19_CLIP-seq_miRNA, and miRTarBase) revealed that miR-155-5p could target the following cell cycle-related and proliferation-related genes: CDK2, CDK4, CCND1, and CCND2.

### 2.9. Gene Ontology (GO) and Kyoto Encyclopedia of Genes and Genomes (KEGG) Enrichment Analysis

DAVID (version 6.8; https://david.ncifcrf.gov/) was used to conduct GO enrichment analysis of miR-155-5p target genes. The top 40 genes were selected for mapping. KOBAS version 2.0 (https://kobas.cbi.pku.edu.cn/) was used to analyze the KEGG enrichment pathway of the top 25 miR-155-5p target genes and associated signaling pathways.

### 2.10. Dual-Luciferase Reporter Gene Assay

pmirGLO-CDK2 3′-untranslated region wild-type (WT) and mutant (mut) plasmids were synthesized by General Biology (Anhui) Co., Ltd. (Chuzhou, Anhui, China). 293T cells were seeded into 24-well plates (5 × 10^5^ cells/well). Cells were cotransfected with WT or mut reporter vector and hsa-miR-155-5p mimics or mimics NC duplexes using Lipo 2000 (Invitrogen, USA). At 48 h after transfection, cell lysates were prepared and a dual-luciferase reporter assay kit (FR201-01; TransGen Biotech, China) was used to measure luciferase activities, according to the manufacturer's instructions. The relative luciferase activities were calculated based on the firefly/renilla luciferase activity ratios.

### 2.11. Statistical Analysis

Statistical analysis was performed using GraphPad version 8.0. Experimental data were expressed as the mean ± standard deviation with at least three replicates. Differences between two or more groups were analyzed using student's *t*-test or one-way analysis of variance. A *P* value of <0.05 was considered to indicate a statistically significant difference.

## 3. Results

### 3.1. Mini-*α*A Inhibits Oxidative Stress-Induced Apoptosis of ARPE-19 Cells

**Figure 1:**
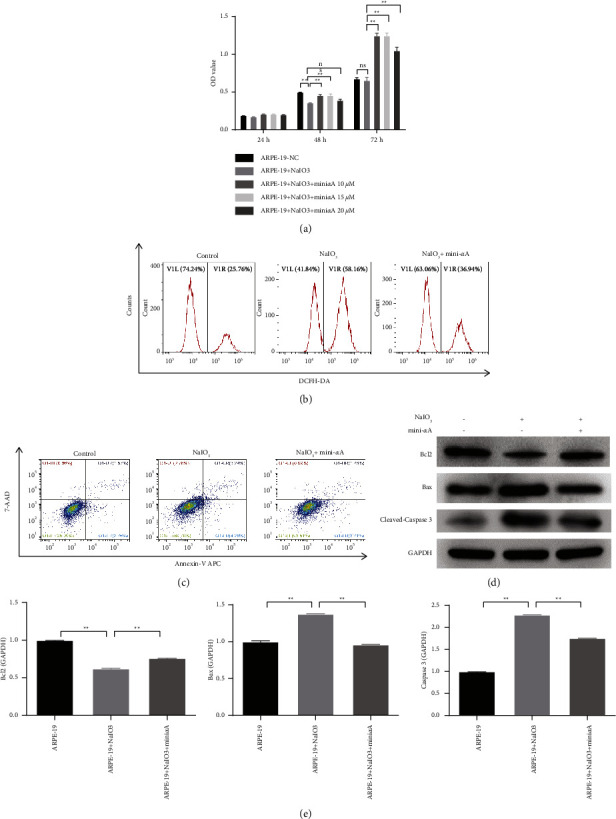
Antiapoptotic effect of mini-*α*A on oxidative stress-induced apoptosis of ARPE-19 cells. ARPE-19 cells were treated with NaIO_3_ to establish a retinal degeneration model, and mini-*α*A was used for treatment; (a) cell viability was examined using CKK-8. (b) ROS level was detected using reactive oxygen species assay kit. (c) Apoptosis was detected using annexin V/7-AAD double staining kit. (d) The expression of Bcl2, Bax, and cleaved caspase-3 was measured using western blotting. (e) Quantitative analysis of protein in (d); ns, not significant; ^*∗∗*^*P* < 0.01.

To determine the role of mini-*α*A during oxidative stress-induced apoptosis, ARPE-19 cells were treated with mini-*α*A. The CCK-8 assay revealed that compared with the control group, cell viability decreased in the NaIO_3_ group and increased in the NaIO_3_ + mini-*α*A group (Figure 1(a)), suggesting that mini-*α*A has a protective effect on the NaIO_3_-induced retinal degeneration model, with 10 *μ*M mini-*α*A exhibiting the most protective effect. Therefore, 10 *μ*M mini-*α*A was selected for subsequent experiments. Additionally, the ROS levels significantly increased in ARPE-19 cells treated with NaIO_3_ for 48 h (Figure 1(b)), which got significantly reduced following the treatment with 10 *μ*M mini-*α*A for 48 h, indicating that mini-*α*A protects ARPE-19 cells from NaIO_3_-induced oxidative damage. Furthermore, flow cytometry analysis revealed that compared with the control group, apoptosis increased in the NaIO_3_ group and decreased in the NaIO_3_ + mini-*α*A group (Figure 1(c)). Western blotting further confirmed that compared with the control group, Bcl-2 expression decreased and Bax and cleaved caspase-3 expression increased in the NaIO_3_ group, whereas Bcl2 expression increased and Bax and cleaved caspase-3 expression decreased in the NaIO_3_ + mini-*α*A group (Figure 1(d) and 1(e)). These results indicate that NaIO_3_ induces oxidative damage and promotes apoptosis in ARPE-19 cells, which were reversed by mini-*α*A.

### 3.2. Screening of miRNA and Bioinformatics Prediction and Analysis of miR-155-5p

**Figure 2:**
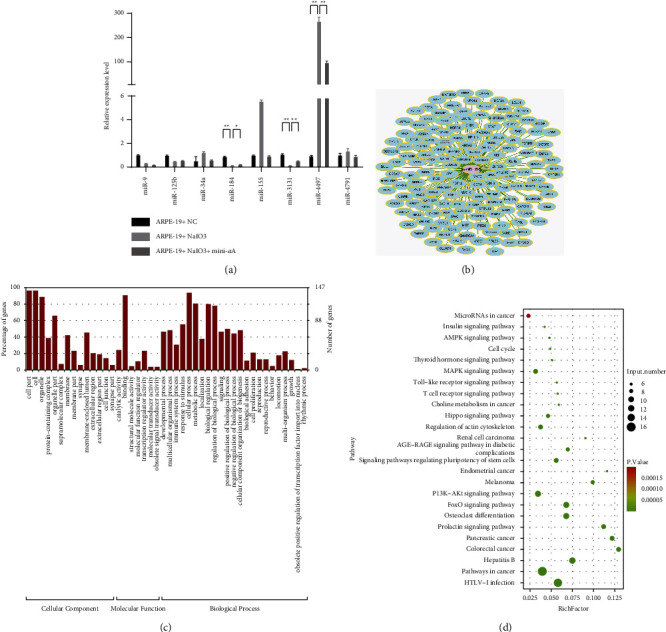
miRNA screening and enrichment analysis of target genes. (a) Eight miRNA (miR-9-5p, miR-125b-5p, miR-34a-5p, miR-184, miR-155-5p, miR-3131, miR-4497, and miR-4491) levels were determined using qRT-PCR. (b) Network map of miR-155-5p target genes. (c) GO analysis diagram of miR-155-5p target genes. (d) KEGG analysis of miR-155-5p target gene. ^*∗*^*P* < 0.05 and ^*∗∗*^*P* < 0.01.

Eight miRNAs (miR-9-5p, miR-125b-5p, miR-34a-5p, miR-184, miR-155-5p, miR-3131, miR-4497, and miR-4491) were screened for verification [9–11]. Among the eight candidate miRNAs, miR-155-5p was significantly upregulated upon NaIO_3_ addition and downregulated after mini-*α*A treatment (Figure 2(a)), suggesting that miR-155-5p plays an inhibitory role in RPE cell apoptosis and can be used as a biomarker in subsequent experiments. Upregulation of miR-155-5p expression in AMD was confirmed by using qRT–PCR; therefore, it was selected for subsequent studies [10].  Figure 2(b) presents the network diagram of miR-155-5p target genes. Through GO analysis, differentially expressed genes were divided into three categories: cellular component (cell part, cell, and organelle), molecular function (binding, catalytic activity, and transcription regulator activity), and biological processes (cellular process, metabolic process, and biological regulation) (Figure 2(c)). KEGG analysis revealed the top 25 signaling pathways, including microRNAs in cancer and cell cycle and pathways in cancer (Figure 2(d)).

### 3.3. Mini-*α*A Plays a Protective Role during Oxidative Damage and Apoptosis Induced by NAIO_3_ by Downregulating miR-155-5p

**Figure 3:**
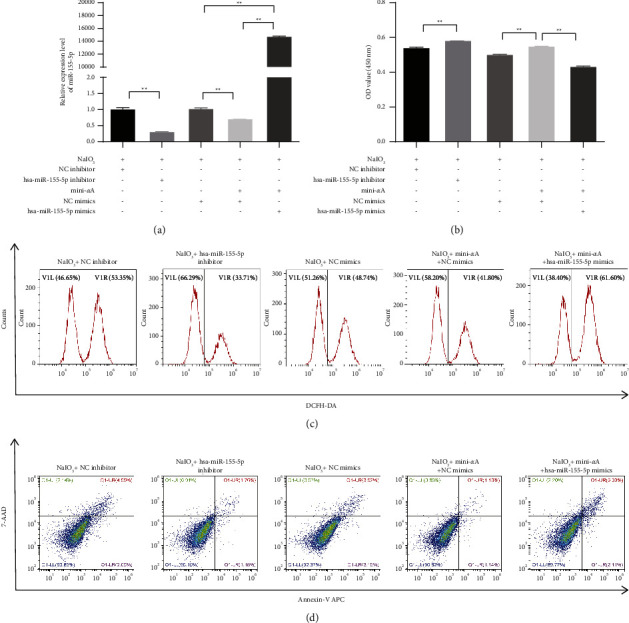
miR-155-5p participates in the therapeutic effect of mini-*α*A on oxidative damage. ARPE-19 cells were treated with NaIO_3_ following which interference and overexpression of miR-155-5p were performed. (a) Interference and overexpression efficiency of miR-155-5p was confirmed by qRT-PCR. (b) Cell viability was determined by CCK-8. (c) ROS level was assessed by reactive oxygen species assay kit. (d) Apoptosis was detected using annexin V/7-AAD double staining. ^*∗*^*P* < 0.05 and ^*∗∗*^*P* < 0.01.

To determine whether miR-155-5p was involved in the therapeutic effect of mini-*α*A on oxidative damage, miR-155-5p interference and overexpression were performed.  Figure 3(a) shows the successful transfection of miR-155-5p constructs. Compared with the NaIO_3_ + NC inhibitor group, cell viability increased and ROS levels and apoptosis rates decreased in the NaIO_3_ + miR-155-5p inhibitor group. Compared with the NaIO_3_ + NC mimics group, cell viability increased and ROS levels and apoptosis rates significantly decreased in the NaIO_3_ + mini-*α*A + NC mimics group. Moreover, cell viability in the NaIO_3_ + mini-*α*A + miR-155-5p mimics group significantly decreased, with a significant increase in the ROS levels and apoptosis rates (Figures 3(b)–3(d)), suggesting that miR-155-5p is involved in the therapeutic effect of mini-*α*A on oxidative damage and apoptosis.

### 3.4. Mini-*α*A Inhibits miR-155-5p Expression and Plays an Antiapoptotic Role by Upregulating Its Target Gene CDK2

**Figure 4:**
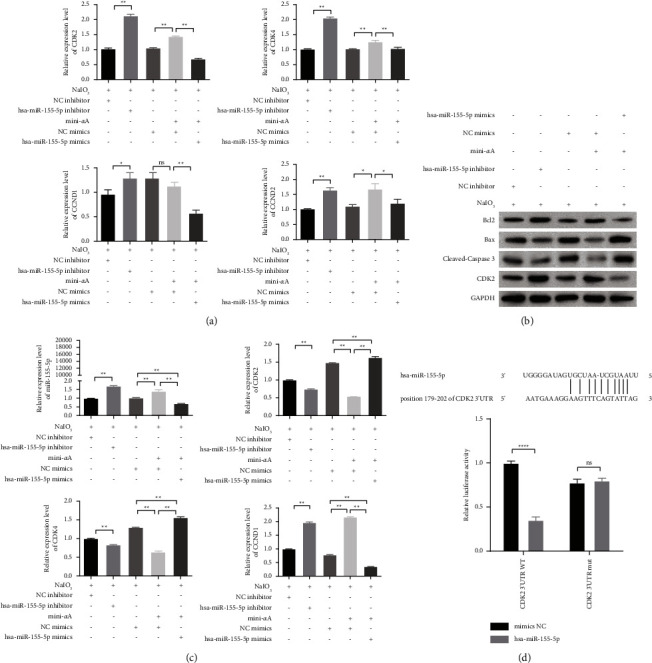
miR-155-5p participates in the therapeutic effects of mini-*α*A by regulating CDK2. ARPE-19 cells were treated with NaIO_3_ following which interference and overexpression of miR-155-5p were performed. (a) Four target genes related to the cell cycle predicted by miRanda were detected by qRT-PCR. (b) The expression of Bcl2, Bax, cleaved caspase-3, and CDK2 was measured by western blotting. (c) Densitometric quantitation of proteins is probed in Figure 4(b). (d) The binding site between 3′UTR of CDK2 and miR-155-5p was predicted by miRanda, and dual-luciferase reporter assay was performed to detect the luciferase activity in wild-type (WT) and mutant (mut) of 293T cells after transfection with NC mimics and miR-155-5p mimics. ns, not significant; ^*∗*^*P* < 0.05 and ^*∗∗*^*P* < 0.01.

Based on various bioinformatic tools (miRPathDB, https://mpd.bioinf.uni-sb.de/mirna.html?mirna=hsa-miR-155-5p&organism=hsa, hg19_CLIP-seq_miRNA, and miRTarBase), we determined that miR-155-5p can target the following cell cycle-related and proliferation-related genes: CDK2, CDK4, CCND1, and CCND2. Therefore, these genes were selected for further analysis. qRT-PCR revealed that compared with the NaIO_3_ + NC inhibitor group, the expression of these four genes was significantly increased in the NaIO_3_ + miR-155-5p inhibitor group (Figure 4(a)). Additionally, compared with the NaIO_3_ + NC mimics group, the expression of CDK2, CDK4, and CCND2, but not CCND1, increased in the NaIO_3_ + mini-*α*A + NC mimics group. Furthermore, compared with the NaIO_3_ + mini-*α*A + NC mimics group, the expression of CDK2, CDK4, CCND1, and CCND2 decreased in the NaIO_3_ + mini-*α*A + miR-155-5p mimics group. Among these genes, CDK2 conformed to the expected change. Subsequently, CDK2 and proteins involved in apoptosis were analyzed using western blotting. Compared with the NaIO_3_ + NC inhibitor group, Bax and cleaved caspase-3 expression decreased and Bcl-2 and CDK2 expression increased in the NaIO_3_ + miR-155-5p inhibitor group. Furthermore, compared with the NaIO_3_ + NC mimics group, Bax and cleaved caspase-3 expression decreased and Bcl-2 and CDK2 expression increased in the NaIO_3_ + mini-*α*A + NC mimics group. Additionally, compared with the NaIO_3_ + mini-*α*A + NC mimics group, Bax and cleaved caspase-3 expression significantly increased and Bcl-2 and CDK2 expression significantly decreased in the NaIO_3_ + mini-*α*A + miR-155-5p mimics group (Figure 4(b) and 4(c)).  Figure 4(d) shows the binding sites of hsa-miR-155-5p and CDK2. Dual-luciferase reporter gene assay also confirmed that CDK2 was the target gene of hsa-miR-155-5p, suggesting that mini-*α*A inhibits RPE cell apoptosis induced by NaIO_3_ through the miR-155-5p/CDK2 axis.

## 4. Discussion

AMD is a common irreversible eye disease characterized by visual impairment in the elderly [24]. RPE cell death caused by oxidative stress plays a vital role in retinal degeneration pathology and is associated with AMD [25]. Therefore, the protection of oxidative stress-induced RPE cells through antioxidative damage and antiapoptotic effects play a crucial role in treating AMD. In this study, we found that inhibiting the expression of miR-155-5p promoted the antiapoptotic effect of mini-*α*A on oxidative stress-induced RPE cell apoptosis via CDK2 regulation. For this purpose, an *in vitro* NaIO_3_-induced retinal degeneration model was established and treated with mini-*α*A, followed by bioinformatics prediction and functional verification.

NaIO_3_ is an oxidative toxic agent and its selective RPE cell damage makes it reproducible in *in vitro* and *in vivo* models of AMD [26]. Although NaIO_3_ is not involved in AMD pathology, it can be utilized to understand the mechanism of RPE cell degeneration [27]. Oxidative stress-induced RPE cell apoptosis is an important pathogenic marker of AMD [28]. Oxidative stress affects the lipid-rich retinal outer segment structure and light processing in the macula [29]. In RPE cells, NaIO_3_-induced oxidative stress coordinates with multiple pathways to induce cell death. For example, kaempferol protects ARPE-19 cells from H_2_O_2_-induced oxidative damage and apoptosis through Bax/Bcl-2 and caspase-3 signaling pathways [30]. *α*-crystallin protects cells from oxidative stress-induced apoptosis [31]. mini-*α*A is derived from a highly conserved region of the human lens protein *α*A-crystallin and exerts anti-inflammatory effects [32]. A previous study reported that mini-*α*A can protect RPE cells from apoptosis induced by NaIO_3_ [20]. Consistently, this study revealed that mini-*α*A can reverse the oxidative damage and apoptosis induced by NaIO_3_ in the retinal degeneration model.

Many regulatory miRNAs have been implicated in AMD pathology and function [33, 34]. Various miRNAs have been proven to be associated with AMD caused by oxidative stress [35, 36]. In this study, eight miRNAs were selected for verification based on the literature to determine their role in AMD [9–11]. Among them, the expression of miR-155-5p was upregulated in cells treated with NaIO_3_ and downregulated in those treated with mini-*α*A. This suggested that miR-155-5p played a significant role in the NaIO_3_-induced RPE cell retinal degeneration model. Further bioinformatics analysis revealed that miR-155-5p can target the following cell cycle-related and proliferation-related genes: CDK2, CDK4, CCND1, and CCND2. Therefore, genes involved in the miR-155-5p-mRNA network can help understand the onset and development of AMD, which warrants further exploration in future studies.

Several studies have reported the role of miR-155-5p in eye-related diseases. For example, toxoplasmosis is associated with miR-155-5p upregulation [37]. During corneal wound healing, miR-155-5p reduces corneal epithelial permeability by reshaping tight epithelial junctions [38]. In diabetic macular edema, the inhibition of miR-155-5p expression downregulates cell proliferation, angiogenesis, and vascular endothelial growth factor levels [39]. These studies demonstrate that miR-155-5p can potentially be used as a biomarker for eye-related diseases. A previous study revealed that decreased miR-1246 expression enhanced the antiapoptotic effect of mini-*α*A on RPE cells during oxidative stress [40]. Moreover, the expression of miR-155-5p was upregulated in the retina of individuals with advanced AMD [10]. Therefore, we interfered and overexpressed miR-155-5p to determine the mechanism of miR-155-5p in the therapeutic effect of mini-*α*A during oxidative damage.

CDK2 belongs to the CDK serine/threonine kinase family and is an important regulator of *G*_1_/*S*-phase conversion. Bevacizumab significantly reduces CDK2, CDK4, and CDK6 as well as cyclin *D* and *E* expression and has a preventive effect on AMD by blocking *G*_1_/*S* progression in ARPE-19 cells [41]. In addition, miR-34a inhibits RPE cell proliferation and migration by downregulating its target CDK2 and other cell cycle-related molecules [12]. This suggests that CDK2 plays a significant role in AMD. Based on bioinformatics prediction and functional validation, we revealed that miR-155-5p may be associated with the antioxidative and apoptotic effect of mini-*α*A via CDK2 regulation. Therefore, miR-155-5p-mediated CDK2 regulation might play a vital role in AMD and could be utilized as a novel molecular biomarker for AMD. However, this study has some limitations, and further studies are warranted to verify the identified miRNA/mRNA role in AMD pathogenesis.

## 5. Conclusion

AMD is a degenerative disease of RPE cells; therefore, determining the role of RPE cells in the disease progression has great clinical significance. NAIO_3_ can induce the degeneration of RPE cells. Our study revealed that mini-*α*A can attenuate the NaIO_3_-induced apoptosis and ROS level elevation in RPE cells and can inhibit NaIO_3_-induced upregulation of miR-155-5p. Interference of miR-155-5p expression in NaIO_3_-induced retinal degeneration cell model reduced cell apoptosis and intracellular ROS levels; moreover, miR-155-5p could target CDK2. In conclusion, miR-155-5p promotes the antiapoptotic role of mini-*α*A in oxidative stress-induced RPE cell apoptosis via CDK2 regulation. This study provides a basis for AMD clinical treatment and prognosis and a novel target for treating AMD.

## Figures and Tables

**Table 1:** The primers used in this study.

Primer ID	5′–3′
Hsa-miR-9-5p-RT	GTCGTATCCAGTGCAGGGTCCGAGGTATTCGCACTGGATACGACTCATAC
Hsa-miR-9-5p-F	AAGCGCCTTCTTTGGTTATCTAG
Hsa-miR-125b-5p-RT	CTCAACTGGTGTCGTGGAGTCGGCAATTCAGTTGAGTCACAAGT
Hsa-miR-125b-5p-F	GCCGAGTCCCTGAGACCCTA
Hsa-miR-34a-5p-RT	GTCGTATCCAGTGCAGGGTCCGAGGTATTCGCACTGGATACGACACAACC
Hsa-miR-34a-5p-F	AACACGCTGGCAGTGTCTTA
Hsa-miR-184-RT	GTCGTATCCAGTGCAGGGTCCGAGGTATTCGCACTGGATACGACACCCTT
Hsa-miR-184-F	GCGTGGACGGAGAACTGAT
Hsa-miR-155-5p-RT	GTCGTATCCAGTGCAGGGTCCGAGGTATTCGCACTGGATACGACAACCCC
Hsa-miR-155-5p-F	TTAATGCTAATCGTGATAGG
Hsa-miR-3131-RT	GTCGTATCCAGTGCAGGGTCCGAGGTATTCGCACTGGATACGACAAGGCC
Hsa-miR-3131-F	TCGAGGACTGGTGGAAGGG
Hsa-miR-4497-RT	GTCGTATCCAGTGCAGGGTCCGAGGTATTCGCACTGGATACGACGCCCAG
Hsa-miR-4497-F	CTCCGGGACGGCTG
Hsa-miR-4791-RT	GTCGTATCCAGTGCAGGGTCCGAGGTATTCGCACTGGATACGACTTTCAG
Hsa-miR-4791-F	TGGATATGATGACTG
CDK2-F	AGCTATCTGTTCCAGCTGCTC
CDK2-R	CAAAGTCTGCTAGCTTGATGGC
CDK4-F	AGCCGAAACGATCAAGGATCT
CDK4-R	GCTCGGTACCAGAGTGTAACA
CCND1-F	GCTGCGAAGTGGAAACCATC
CCND1-R	CCTCCTTCTGCACACATTTGAA
CCND2-F	CAAGCATGCTCAGACCTTCA
CCND2-R	AGCTTTGAGACAATCCACGTCT
GAPDH-F	GAGTCAACGGATTTGGTCGT
GAPDH-R	GACAAGCTTCCCGTTCTCAG
U6-F	CTCGCTTCGGCAGCACA
U6-R	AACGCTTCACGAATTTGCGT

## Data Availability

The datasets used and/or analysed during the current study are available from the corresponding author on reasonable request.

## References

[1] Simó R., Villarroel M., Corraliza L., Hernandez C., Garcia-Ramirez M. (2010). The retinal pigment epithelium: something more than a constituent of the blood-retinal barrier--implications for the pathogenesis of diabetic retinopathy. *Journal of Biomedicine and Biotechnology*.

[2] Hanus J., Anderson C., Wang S. (2015). RPE necroptosis in response to oxidative stress and in AMD. *Ageing Research Reviews*.

[3] Wong W. L., Su X., Li X. (2014). Global prevalence of age-related macular degeneration and disease burden projection for 2020 and 2040: a systematic review and meta-analysis. *Lancet Global Health*.

[4] Le Y. Z. (2017). VEGF production and signaling in Müller glia are critical to modulating vascular function and neuronal integrity in diabetic retinopathy and hypoxic retinal vascular diseases. *Vision Research*.

[5] Mitchell P., Liew G., Gopinath B., Wong T. Y. (2018). Age-related macular degeneration. *The Lancet*.

[6] Lu Z. G., May A., Dinh B. (2021). The interplay of oxidative stress and ARMS2-HTRA1 genetic risk in neovascular AMD. *Vessel Plus*.

[7] Totsuka K., Ueta T., Uchida T. (2019). Oxidative stress induces ferroptotic cell death in retinal pigment epithelial cells. *Experimental Eye Research*.

[8] Blasiak J., Watala C., Tuuminen R. (2019). Expression of VEGFA-regulating miRNAs and mortality in wet AMD. *Journal of Cellular and Molecular Medicine*.

[9] Berber P., Grassmann F., Kiel C., Weber B. H. F. (2017). An eye on age-related macular degeneration: the role of microRNAs in disease pathology. *Molecular Diagnosis and Therapy*.

[10] SanGiovanni J. P., SanGiovanni P. M., Sapieha P., De Guire V. (2017). miRNAs, single nucleotide polymorphisms (SNPs) and age-related macular degeneration (AMD). *Clinical Chemistry and Laboratory Medicine*.

[11] Romano G. L., Platania C. B. M., Drago F. (2017). Retinal and circulating miRNAs in age-related macular degeneration: an in vivo animal and human study. *Frontiers in Pharmacology*.

[12] Hou Q., Tang J., Wang Z. (2013). Inhibitory effect of microRNA-34a on retinal pigment epithelial cell proliferation and migration. *Investigative Ophthalmology & Visual Science*.

[13] Phadte A. S., Sluzala Z. B., Fort P. E. (2021). Therapeutic potential of *α*-crystallins in retinal neurodegenerative diseases. *Antioxidants*.

[14] Pasupuleti N., Matsuyama S., Voss O. (2010). The anti-apoptotic function of human *α*A-crystallin is directly related to its chaperone activity. *Cell Death & Disease*.

[15] Yaung J., Jin M., Barron E. (2007). alpha-Crystallin distribution in retinal pigment epithelium and effect of gene knockouts on sensitivity to oxidative stress. *Molecular Vision*.

[16] Sreekumar P. G., Spee C., Ryan S. J., Cole S. P. C., Kannan R., Hinton D. R. (2012). Mechanism of RPE cell death in *α*-crystallin deficient mice: a novel and critical role for MRP1-mediated GSH efflux. *PLoS One*.

[17] Mao Y. W., Liu J. P., Xiang H., Li D. W. C (2004). Human *α*A- and *α*B-crystallins bind to Bax and Bcl-XS to sequester their translocation during staurosporine-induced apoptosis. *Cell Death & Differentiation*.

[18] Bhattacharyya J., Sharma K. K. (2001). Conformational specificity of mini-*α*A-crystallin as a molecular chaperone. *The Journal of Peptide Research*.

[19] Sreekumar P. G., Chothe P., Sharma K. K. (2013). Antiapoptotic properties of *α*-crystallin-derived peptide chaperones and characterization of their uptake transporters in human RPE cells. *Investigative Ophthalmology & Visual Science*.

[20] Zhang J., Zhao X., Cai Y., Li Y., Yu X., Lu L. (2015). Protection of retina by mini-*α*A in NaIO3-induced retinal pigment epithelium degeneration mice. *International Journal of Molecular Sciences*.

[21] Enzbrenner A., Zulliger R., Biber J. (2021). Sodium iodate-induced degeneration results in local complement changes and inflammatory processes in murine retina. *International Journal of Molecular Sciences*.

[22] Raju M., Santhoshkumar P., Henzl T. M., Sharma K. K. (2011). Identification and characterization of a copper-binding site in *α*A-crystallin. *Free Radical Biology and Medicine*.

[23] Chen Q., Lin H., Deng X., Li S., Zhang J. (2020). MiR-1246 promotes anti-apoptotic effect of mini-*α*A in oxidative stress-induced apoptosis in retinal pigment epithelial cells. *Clinical and Experimental Ophthalmology*.

[24] Ruan Y., Jiang S., Gericke A. (2021). Age-related macular degeneration: role of oxidative stress and blood vessels. *International Journal of Molecular Sciences*.

[25] Chen S. J., Lin T. B., Peng H. Y. (2021). Cytoprotective potential of fucoxanthin in oxidative stress-induced age-related macular degeneration and retinal pigment epithelial cell senescence in vivo and in vitro. *Marine Drugs*.

[26] Chan C. M., Huang D. Y., Sekar P., Hsu S. H., Lin W. W. (2019). Reactive oxygen species-dependent mitochondrial dynamics and autophagy confer protective effects in retinal pigment epithelial cells against sodium iodate-induced cell death. *Journal of Biomedical Science*.

[27] Tong Y., Wang S. (2020). Not all stressors are equal: mechanism of stressors on RPE cell degeneration. *Frontiers in Cell and Developmental Biology*.

[28] Xie R., Wang B., Zuo S. (2022). Protective effects of CRTH2 suppression in dry age-related macular degeneration. *Biochemical and Biophysical Research Communications*.

[29] Blasiak J., Petrovski G., Vereb Z., Facsko A., Kaarniranta K. (2014). Oxidative stress, hypoxia, and autophagy in the neovascular processes of age-related macular degeneration. *BioMed Research International*.

[30] Du W., An Y., He X., Zhang D., He W. (2018). Protection of kaempferol on oxidative stress-induced retinal pigment epithelial cell damage. *Oxidative Medicine and Cellular Longevity*.

[31] Askou A. L., Alsing S., Holmgaard A., Bek T., Corydon T. J. (2018). Dissecting microRNA dysregulation in age-related macular degeneration: new targets for eye gene therapy. *Acta Ophthalmologica*.

[32] Muralidharan A., Tender T., Shetty P. K., Mutalik S., Hariharapura R. C. (2021). Anti-inflammatory activity of human lens crystallin derived peptide. *Current Drug Delivery*.

[33] ElShelmani H., Wride M. A., Saad T., Rani S., Kelly D. J., Keegan D. (2020). Identification of novel serum microRNAs in age-related macular degeneration. *Translational Vision Science & Technology*.

[34] Peplow P., Martinez B. (2021). MicroRNAs as diagnostic and prognostic biomarkers of age-related macular degeneration: advances and limitations. *Neural Regen Res*.

[35] Haque R., Chun E., Howell J. C., Sengupta T., Chen D., Kim H. (2012). MicroRNA-30b-mediated regulation of catalase expression in human ARPE-19 cells. *PLoS One*.

[36] Ayaz L., Dinç E. (2018). Evaluation of microRNA responses in ARPE-19 cells against the oxidative stress. *Cutaneous and Ocular Toxicology*.

[37] Meira-Strejevitch C. S., Pereira IdS., Hippolito D. D. C. (2020). Ocular toxoplasmosis associated with up-regulation of miR-155-5p/miR-29c-3p and down-regulation of miR-21-5p/miR-125b-5p. *Cytokine*.

[38] Wang F., Wang D., Song M., Zhou Q., Liao R., Wang Y. (2020). miRNA-155-5p reduces corneal epithelial permeability by remodeling epithelial tight junctions during corneal wound healing. *Current Eye Research*.

[39] He J., Zhang R., Wang S. (2021). Expression of microRNA-155-5p in patients with refractory diabetic macular edema and its regulatory mechanism. *Experimental and Therapeutic Medicine*.

[40] Hwang N. (2019). Oxidative stress-induced pentraxin 3 expression human retinal pigment epithelial cells is involved in the pathogenesis of age-related macular degeneration. *International Journal of Molecular Sciences*.

[41] Kuo C. N., Chen C. Y., Lai C. H. (2012). Cell cycle regulation by bevacizumab in ARPE-19 human retinal pigment epithelial cells. *Molecular Medicine Reports*.

